# Liberty of Medical Opinion and Action

**Published:** 1881-04

**Authors:** 


### HOMŒOPATHIC PHYSICIAN.

A MONTHLY JOURNAL OF MEDICAL SCIENCE.

"If our school ever gives up the strict inductive method of Hahnemann, we are lost, and deserve to be mentioned only as a caricature, in the history of medicine." — CONSTANTINE HERING.

Vol. I. APRIL, 1881. No. 4.

### EDITORIAL.

Liberty of Medical Opinion and Action.—It is often profitable for us to study the writings and practice of those who are, or have been, noted for their learning, ability and skill. In this spirit, we propose to briefly examine, as to a few points, some essays of the late Dr. Carroll Dunham.

At the Milwaukee meeting of the Am. Inst. of Homœopathy, a member declared that Dr. Dunham's "ringing words, proclaiming absolute liberty of medical opinion and action, which stand upon our record like letters of gold, have done more to advance homœopathy—true homœopathy—than those of any other man that have ever been spoken."* It is this claim which we wish to consider; trying fairly to ascertain what Dr. Dunham meant by this declaration and to see what has been its effect upon the homœopathic school.

In this speech Dr. Dunham said:

"Mr. President, and my colleagues! my own position on these

* Taken from Milwaukee *Sentinal*, of June 17, 1880.

points of doctrine is not unknown to some of you. Holding that the law *Similia Similibus Curantur*, expresses the relation between the specific drug action and the diseased organism, and that it is a sufficient, and the only trustworthy guide in every application of drugs to cure the sick, I fully believe not only that the practical rules laid down by Hahnemann, and which enjoin the single remedy and the minimum dose, are the rules of sound practice, but I believe that every *observing physician who faithfully applies the law of cure*,* will be led by experience to the same conclusion, and will adopt these rules as leading to the best results. Notwithstanding this belief, I advocate entire liberty of opinion and practice. Nay, *because* of this belief, I plead for liberty; for I am sure perfect liberty will the sooner bring knowledge of the truth and that purity of practice which we all desire."†

"Every observing physician who faithfully applies the law of cure" will be convinced, says Dr. Dunham. To this, all true homœopaths agree. In that way were Bœnninghausen, Gross, Stapf, Hering, Dunham, and a host of other genuine homœopaths converted. Was any one ever converted to homœopathy who did not apply the *law faithfully?* Dr. Dunham evidently believed this impossible; for he explicitly states that "every observing physician who faithfully applies the law" will be convinced. It is not only necessary that he be an observing physician, he must also apply *the* law (that is, the *one* true law) faithfully (that is, as Hahnemann directs) or he will fail to obtain the "best results." This argument of Dr. Dunham's excludes all those who do not faithfully apply the law; it excludes all those who combine or alternate remedies; it excludes all who palliate or purge; it excludes all those who practice contrary to the logic of "the law" and who teach such deviations as right and necessary. These persons do not apply the law at all, nor understand its first principles; one of this class of "philosophical physicians," as Hempel styled them, denies that he "sees one particle of proof that the dynamized remedy given singly and in the smallest dose....is alone homœopathic;" also says "the man who never uses topical applica-

* Italics are ours.—Ed.

† *North Americrn Journal of Homœopathy*, vol. xix., p. 115.

tions or mechanical appliances in non-surgical cases is unfaithful to his patients." This individual we quote as a fair sample of those who *never applied the law faithfully and hence failed to obtain* "the best results." They never were convinced, never will be convinced, and never want to be convinced, of its truth. They simply desire to have Dr. Dunham's sanction to use the name of homœopath as a trade mark. This sanction we can prove Dr. Dunham never gave.

The object of association and fellowship is to mutually improve one another by an interchange of knowledge and experience. It is evident that a believer in "the law" could not learn from one who did not believe in, or know aught of that law, for the one would differ from the other too widely in therapeutics and pathology; nor could he, who would not honestly and faithfully apply that law, gain the "best results," and be convinced; therefore it follows that such fellowship would never be congenial or profitable. A sensible man like Dr. Dunham could never advocate fellowship with such uncongenial company. No! it was in behalf of the honest inquirer, for those who saw as yet but dimly, but who *desired* earnestly to know and see the truth more clearly, that Dr. Dunham said: "let us bear with them, assist them, teach them." Being himself diligent, he believed all physicians were diligent; being himself honest, he believed all physicians were honest; hence he said, bear with these weak brethren a little longer and they will become true and conscientious healers; will become able and honest homœopaths. 'Twas not to those whose laziness these "ringing words" now shield, nor to ignorance, which seeks to pass as liberty of opinion, nor to dishonesty, which seeks to masquerade as liberty of action, that he spake! To none of these had Carroll Dunham aught to say, save to repeat *Hahnemann's* "ringing words," saying: "in a science in which the welfare of mankind is concerned, any neglect to make ourselves masters of it is a *crime*."*

* See, *North American Journal of Homœopathy*, vol. xix, p. 124.

Let us now see what Dr. Dunham thought of the various departures from the strict inductive method of Hahnemann, now so fashionable, and which Dr. Hering declared would cause such deserters to be considered hereafter as mere caricatures of physicians. We make a few quotations from his various papers, that it may be clearly seen how Dr. Dunham denounced such doings.

Alternation of Remedies. — Of this heresy Dr. Dunham declared, "the requirements of a sound homœopathic prescription can not be met by the process of alternation."* In order to leave no doubt as to what he considered a sound homœopathic prescription, he further says:

"*First.*—It requires that before every prescription, the symptoms of the patient shall be studied anew.... We have seen that in the ordinary method of alternation (*a priori*) this is not attempted to be done and can not be done; it is not proposed to do it.

"*Secondly.*—It requires that the aggregate of the symptoms presented by the patient be regarded as one malady, for which an analogue is to be found in the Materia Medica." He adds, (as if to give, at one stroke, a death blow to the twin heresies, of combination and alternation) "We have no authority in science for arbitrarily dividing this aggragate of symptoms into groups, for each group of which we are to find an analogue in the Materia Medica, and then giving these analogues in combination or alternation.

"*Thirdly.*—It requires that a drug shall be selected which has produced on the healthy subject, symptoms very similar to those of the patient.... If drugs had been proved in alternation, we might then with propriety, *perhaps*, prescribe them in alternation. Until this is done, the method is a hap-hazard, chance operation."†

On another occasion, speaking against this same "crime," he

---

* See, "Homœopathy, the Science of Therapeutics," p. 192.

† Ibid, p. 183, et. sq.

says, "we feel driven to the conclusion that, if excluded from the ground of *scientific principle*, we have no ground left on which to stand for the discussisn of this or any other question of medical practice; further than this we have nothing to say."*

Here we find Dr. Dunham taking an advanced stand in favor of inductive science; so much so that he declares he does not even care to discuss practices which are not based on scientific principles! Can this man be fairly quoted as authorizing alternation, an unscientific practice? We think not.

PATHOLOGICAL PRESCRIBING.—And what were Dr. Dunham's views as to pathological prescribing? Do we find him agreeing with those who claim that "Scientific therapeutics consists in more than mere symptom hunting"? Not at all! He says: "Physiology and pathology themselves teach us that the science of pathology can in no sense serve as a basis or foundation for the science of therapeutics." Again: "But these advances in pathology, great as they have been, have not altered the relation which the phenomena of natural disease bear to those of drug disease. These phenomena respectively, whether rudely apprehended, or clearly and fully understood in all their relations and inter-dependencies, still bear the same relation to each other expressed by the law *Similia Similibus Curantur*. And we can imagine no possible development of the sciences of pathology and pathogenesy which could alter this relation."† On another occasion he wrote: "And those of our school who insist upon pathology as a *basis* of therapeutics, who look upon the single objective symptom and its nearest organic origin as the subject for treatment, and who deride the notion of prescribing upon the totality of the symptoms, and claim to be more than mere symptom-coverers, in that they discover and aim to remove the *cause* of the disease—these colleagues are as false in their pathology, according to the highest old school authorities, as they are faithless to the doctrines, and impotent

* Ibid, p. 192.

† Ibid, p. 28.

as to the successes of the founder of the homœopathic school"!† To Hahnemann's views on pathology, at which lesser minds now fling their petty ridicule, Dr. Dunham paid the following glowing tribute: "I can not refrain, in conclusion, from rendering homage to that wonderful prevision of genius by which, in an age when pathology, as we understand it, was unknown, Samuel Hahnemann anticipated all that we have said, and all that the most advanced writers of our day have taught respecting the scope and influence of pathology in relation to therapeutics."*

From these few quotations, the candid reader will be able to see just what Dr. Dunham thought of those erroneous practices which are now being perpetrated under the sanction of his name. We conld give many more quotations, from this able writer, all of the same tenor, and equally condemnatory of these practices; but we have *failed*, after prolonged search, to find one sentence authorizing eclectic methods. These quotations, few though they be, prove that Dr. Dunham was in favor of pure Hahnemannian homœopathy; that he did not desire even to discuss practices not based on scientific principles; that he considered the modern departures from strict homœopathy to be unscientific, and impotent as to success. His scientific spirit leads him so far that, while exposing the fallacies of alternation he not only disclaims its use for himself but also for others, saying: "Indeed, in *theory* and in *practice* Dr. Bœnninghausen is as decidedly opposed to alternation as we have shown that Hahnemann was." Both Hahnemann and Bœnninghausen having been *misquoted* by the advocates of lazy alternation, Dr. Dunham thinks it his duty to disprove the assertion. As Dr. Dunham said,

"There are among those who call themselves homœopathists, some who are impostors; men unworthy to be called physicians; men without knowledge and without conscience, who play upon the credulity of mankind, and pervert to their own aggrandize-

† Ibid, p, 114.
* Ibid, p. 113.

ment every trust committed to them. That such men, professing to be of our school, should be regarded by the community as belonging to it, and should tarnish our fair name by their foul deeds, is certainly a misfortune."*

No severer arraignment of these *philosophers* could be penned by the most bitter "Hahnemannian" of to-day. It is this "misfortune" so graphically described by Dr. Dunham, which the strict Hahnamannians wish to avoid. Association with these persons means more than mere instruction, it means endorsement and entails a befogment of the laity so great that they can not separate the wheat from the tares, the homœopath from the mongrel. "But if we do not teach these erring brethren they will continue in their ignorance," says an objector; nay, friend, not so; they have the same source of information that you and I had, the "Organon" of Hahnemann. The "observing physician who faithfully applies the law of cure" will be convinced, says Dr. Dunham. Not he who follows my practice, or your teaching. Homœopathy allows no *ipse dixits*; *that* is allopathy's method, and we envy it not. Follow the law, *the law* is Dr. Dunham's teaching! Those homœopaths who will not study the "Organon" of Samuel Hahnemann from a desire to test its merits will never adopt our law though a thousand physicians trumpet its virtues. To all observing physicians who endeavor to "faithfully" apply the law of cure in order to learn if it be true or false, to all such the strict Hahnemannians offer fellowship, assistance, encouragement, knowing full well that he who does so apply the "law of cure" must adopt that "purity of practice which we all desire." But the alternater of drugs does not faithfully apply the law of cure, neither does the administrator of drugs in crude doses, of palliatives, of tonics, etc., hence, according to Dr. Dunham, these men will never adopt that "purity of practice which we all desire."

They may indeed *claim* "as much success in their treatment

* See, *North American Journal of Homœopathy*, vol. xix, p. 114.

as the most strict Hahnemannian obtains,"† but they can not fairly claim Carroll Dunham as their exemplar and defender; for we find him, in theory and in practice, always, the true disciple of the "strict inductive method of Hahnemann"; always the ardent advocate of the law of the similars, of the single remedy, and the minimum dose, and, *ex necessitate*, always the foe of the modern useless and erroneous departures from that law and those principles.

To be able to cite one sentence only, as authorizing these practices, one sentence from a life's writings, teaching and work is of itself, the confession of a weak and indefensible position. Can these gentlemen mention one occasion where Dr. Dunham approves or tolerates any mixed or eclectic practice? Can they narrate cases so treated by him? Unless they can do these things they must cease quoting him as their exemplar, guide and defender. Or they cease to be true friends of their departed colleague, and instead prove enemies to his fair name and honored reputation.

They glibly quote the beautiful Latin maxim, "*De mortuis nihil nisi bonum*," while degrading his name by connecting it with practices he abhorred. By attempting to shield all manner of eclectic practices behind the noble reputation of Dr. Dunham his inconsiderate or pretended friends do wrong to his memory, in that they insult his honesty and intelligence.

† Ibid for Feb. 1881, p. 528.

